# Saudi Arabian students in postgraduate dental programs: Investigating factors associated with burnout

**DOI:** 10.12688/f1000research.111000.2

**Published:** 2022-11-17

**Authors:** Amal Asiri

**Affiliations:** 1 Dental Education Department, College of Dentistry, Imam Abdulrahman bin Faisal University, Dammam, 31441, Saudi Arabia

**Keywords:** Burnout, health care providers, postgraduate residents, emotional exhaustion, depersonalization, personal accomplishment, Maslach burnout inventory, dentistry.

## Abstract

**Background:** Burnout related to emotional and physical study or work demands affects an individual’s performance and wellbeing. This study focused on Saudi Arabian dental residents studying in the United States and the United Kingdom who are faced with many challenges in the pursuit of a higher education degree.

**Methods**: A survey including demographic and Maslach Burnout inventory (MBI) questions was distributed to assess this population’s level of burnout. The MBI has been widely used in the literature to assess three components of burnout: emotional exhaustion (EE), depersonalization (DEP), and (diminished) personal accomplishment (PA). Potential predictors of burnout level, tested for statistical significance, included: (1) country (US vs UK), (2) hours of work, (3) sponsorship status, (4) marital status (5) gender, and (6) prior work experience.

**Results:** The number of participants from the UK was 29 and from the US was 64 (n=93). The total number of participants who completed the survey was 87. Using multiple regression analyses, those found to predict EE included hours of work, sponsorship status, and gender. Only gender was found to predict PA. None of the variables were predictive of DEP. Moreover, after controlling for the demographic variables, the country where studying did not help account for the level of burnout.

**Conclusion:** In the literature, burnout was found to be prevalent among dental postgraduate students. This study suggests that Saudi Arabian dental residents experience high burnout due to emotional exhaustion when compared to other medical professionals. Moreover, Female residents are more likely to report experiencing burnout than their male counterparts. Limitations of the study, implications for practice and suggestions for further research are offered in the discussion.

## Introduction

Dental students experience high levels of stress due to the nature of their studies and profession. In the US, a dental student typically spends three to four years in dental school following a three-four year bachelor’s degree in a related major. In Saudi Arabia, dental school is a five-year commitment preceded by one preparatory year and ending with one year of internship in public clinics. After passing licensure exams and graduation, some graduates choose to pursue higher education. They compete for acceptance in a variety of postgraduate dental specialty programs where they continue their studies for a few more years. Table 1 illustrates different dental program timelines according to program location. The dental curriculum demands intense understanding of detailed information across an array of disciplines. Requirements include dental laboratory duties, managing of a variety of routine and unpredictable clinical situations, and multiple written, clinical, and licensing examinations throughout dental school years. Numerous studies have indicated that the rigorous training requisites, as well as the span of competencies across clinical, theoretical, and interpersonal requirements, increase rates of stress and burnout in postgraduate dental students. ^1^^–^^5^ Furthermore, there has been an increase in the number of international dental students in postgraduate programs, in which international students can experience additional stressors relating to socio-cultural adjustment, self-efficacy, educational gaps, and other challenges. ^6^^,^^7^

**Table 1.** Breakdown of dental studies timelines according to location in Saudi Arabia, the United States (US), and the United Kingdom (UK).

Country	Pre-dental school	Dental school	Qualification procedures	After graduation options
Saudi Arabia	NA	Six years + one year internship	Saudi Licensing Exam (SLE)	Practice dentistry and/or apply for a postgraduate specialty program
The United States (US)	Pre-dental bachelor’s degree	Three to four years	The National Board Dental Examination (NBDE) + one of the following exams depending on the desired location of practice: The Central Regional Dental Testing Service (CRDTS), Council of Interstate Testing Agencies, Inc. (CITA), the Commission on Dental Competency Assessments (CDCA) [formerly the North East Regional Board of Dental Examiners, Inc./NERB], Southern Regional Testing Agency (SRTA), and Western Regional Examining Board (WREB)	Same as above
The United Kingdom (UK)	NA	Five years + one to two years of dental foundation training	Registration in the General Dental Council (GDC) ^18^	Same as above

Stress is a broad term that can be defined in several ways, though it generally pertains to an individual being overburdened or pressured by the requirements of a given environment, to the point where wellbeing is compromised. ^1^^,^^3^ Burnout was defined as “a psychological syndrome emerging as a prolonged response to chronic interpersonal stressors on the job”. ^8,p.103^ Persistent exposure to stress can lead to burnout, which involves work or school-related exhaustion and disengagement. ^4^ The reported burnout rate among health care providers ranges from moderate to high levels, which implies that health care workers are at a higher risk for job-related burnout than the general population or non-health care providers. ^8^ Studies have found that in addition to the workload itself, dental students experience fatigue, chronic sleep deprivation, an increased likelihood of being on-call, as well as stressors relating to debt accrued from the high costs of education and unsteady financial standing. ^9^

Studies have shown that stress perceived by dental students increases, on average, as students’ progress in dental school, with seniors reporting higher stress and burnout compared to first and second year dental students. ^3^ A study by Harrison *et al.,* ^10^ employed a sample of students at a US dental school where participants were assessed with the Perceived Wellness survey, the Mental Health inventory, and the Medical Outcomes Study Social Support survey. The results indicated that first-year students reported less social support, and that Asian and Hispanic students indicated less happiness and mental wellbeing compared to the other ethnic subgroups. Notably, whether students were international was not factored in this study. The researchers suggested the need for more wellbeing support in dental programs to address varying student needs. ^10^ As dental students are increasingly enrolling in clinical and non-clinical specialty post-graduate programs, either soon after graduation or elsewise during their career, added stressors can hinder wellbeing. ^6^

While stress and burnout related to the study and practice of dentistry in the predoctoral level has been widely studied, the literature is relatively sparse when it comes to stress in post-graduate dental students and residents. Furthermore, literature that specifically assesses international students in postgraduate dental programs is lacking. The average length of a post graduate program is two-three years depending on sought credentials and specialty. Program requirements vary in different countries, and coping mechanisms also vary across different cultures. Three scales of burnout are commonly used in the literature to characterize it: 1) Emotional Exhaustion which measures feelings of being emotionally overextended and exhausted by one's work. 2) Depersonalization which measures an unfeeling and impersonal response toward patients. 3) Personal Accomplishment which measures feelings of competence and successful achievement in one's work. ^11^ Divaris *et al.,* ^12^ assessed students in a postgraduate dental program in Greece. The Graduate Dental Environmental Stress questionnaire was administered to measure stress, and the Maslach Burnout Inventory (MBI) measured burnout, specifically on the scales of personal accomplishment, emotional exhaustion, and depersonalization. On all three scales of the MBI survey, there was a positive correlation of perceived stress and burnout where most of the sample (44%) were deemed burnout cases on the emotional exhaustion scale, followed by personal accomplishment (38%) and depersonalization (13%). Furthermore, perceived stress was prominent among clinical students compared to non-clinical and PhD programs. ^12^ Similarly, when assessing stress and burnout in Swiss dental residents applying the same instruments, it was found that insufficient leisure time, as well as curriculum and research requirements, were among the top stressors. ^4^ The specific requirements in a dental program have considerable effects on students’ capacity to take on the workload, cope with stress, and reduce risks of burnout.

To compare stress and burnout in undergraduate and postgraduate dental students, a study by Mandava *et al.,* ^13^ assessed students across five regions in India. The questionnaire to measure stress was formulated by the International Stress Management Association including the Maslach Burnout Inventory. Results indicated no statistical significance correlating stress and burnout in undergraduate students; however, there was a significant correlation between stress and burnout among postgraduate orthodontic students. Emotional exhaustion and depersonalization were positively correlated with perceived stress measured by the International Stress Management Association questionnaire, as also supported by Divaris *et al.,* ^12^ using the Graduate Dental Environment Stress (GDES). However, personal achievement was negatively correlated with burnout. ^13^

Studies addressing stress and burnout in the Saudi dental residents population are especially lacking or limited to dental residents in the same specialty or within one institution. A study by Al-Sowygh ^14^ assessed perceived stress among undergraduate dental students at a Saudi Arabian dental program with the Dental Environmental Stress questionnaire and found that clinical requirements correlated with the highest stress levels, particularly among fourth and fifth-year students. Notably, ‘social stressors’ and ‘performance pressure’ were heightened among married students compared to single students. ^14^ However, a study by Al-Shayea ^15^ assessed perceived anxiety, depression, and stress among postgraduate orthodontic students across three institutions in Saudi Arabia. The researchers found that married students, and students over the age of 30, indicated lower levels of anxiety, though stress was persistent across all categories and institutions. This study employed the Depression Anxiety Stress Scale which indicated the prevalence of moderate stress levels in the participants. ^15^ This indicates the general pervasiveness of stress in postgraduate programs, contrary to more varied results when assessing undergraduate dental students. Saudi Arabian dental residents studying abroad potentially undergo added stress due to relocating to a new country and culture and moving away from family and home for a prolonged period of time. The aim of this study is to assess burnout and stress levels in Saudi Arabian dental residents enrolled in dental specialty postgraduate programs in the United Kingdom and the United States. Furthermore, this assessment will provide data for comparison between the MBI means of dental residents and medical professionals. This analysis was conducted to shed some light on possible contributory factors that residents might want to take into consideration when planning to study abroad.

### Research questions

This research will address the following research questions: 1) What level of burnout, on average, do Saudi Arabian students enrolled in postgraduate programs report as measured by the emotional exhaustion (EE), depersonalization (DP), and (diminished) personal accomplishment (PA) subscales of the Maslach Burnout Inventory (MBI)?; 2) What proportion of variation in levels of burnout among Saudi Arabian students enrolled in postgraduate programs is accounted for by the set of predictors which includes: country where studying, hours of work, being sponsored or not, marital status, and prior work experience?; and, 3) Which of the following predictors accounts for unique variation in levels of burnout among these students? The predictors are (1) country (US vs UK), (2) hours of work, (3) sponsorship status, (4) marital status (5) gender, and (6) prior work experience. These questions will be assessed with a quantitative comparative research design assessing Saudi students enrolled in postgraduate dental programs in the UK and the US using the Maslach Burnout Inventory - Human Services Survey for Medical Personnel (MBI-HSS MP), ^16^ which is a variation of the MBI tool adapted for medical personnel to measure burnout by addressing three scales: 1) feelings of emotional exhaustion due to work duties, 2) depersonalization measuring lack of sympathy and impersonal attitudes toward patients, and 3) personal accomplishment measuring feelings about success and achievement related to the profession.

### Research significance

Program designs have a significant impact on stress and burnout in postgraduate dental students, and studies indicate that more individualized support for students is needed to reduce stress and burnout. ^1^ Different approaches have been applied in an effort to reduce risks of stress and burnout. Ahmed *et al.,* ^1^ compared a case-based curriculum and a subject-based curriculum for fifth and sixth-year dental and medical students at Kuwait University and found the case-based format correlated with higher stress levels. Some of the leading sources of stress were related to inconsistent feedback from varying instructors, challenges in effective communication with instructors, and workload amount. ^1^ Factors relating to inconsistent feedback, communication challenges are likely to be heightened for international students, in addition to gaps in knowledge and standards in US programs compared to the home country. ^6^ The American Dental Education Association specifies guidelines for robust learning environments for diverse students, as well as measures to address linguistic and cultural backgrounds. ^17^ However, it is unclear what measures are generally applied to address stress in postgraduate programs. To the researchers’ knowledge, there has yet to be a study comparing the stress and burnout among a specific international population in postgraduate dental programs in the United States and the United Kingdom. The study was designed to inform Saudi students about possible predictors of burnout when planning on studying abroad and can also guide US and UK. postgraduate programs in regard to addressing stress and burnout considerations for international students.

### Definition of terms

The terms used in this article pertain to the psychological experiences of burnout and stress. The author also clarifies in this section the elaborate educational requirements for a dental student in the locations of interest of this study. This clarification highlights the educational journey of postgraduate dental residents leading to their enrollment in residency programs in the US or UK. The educational timeline for the US and Saudi Arabia were acquired based on the author’s experience of studying in both countries. The UK dental educational timeline was acquired from the General Dental Council (GDC), the licensing body for dentists practicing in the UK. ^18^

*Burnout:* According to Maslach and Leiter, ^8^ burnout is a psychological syndrome emerging as a prolonged response to chronic interpersonal stressors on the job. The three key dimensions of this response are an overwhelming exhaustion, feelings of cynicism and detachment from the job, and a sense of ineffectiveness and lack of accomplishment.

*Emotional Exhaustion:* measures feelings of being emotionally overextended and exhausted by one's work. ^11^

*Depersonalization:* measures an unfeeling and impersonal response toward patients. ^11^

*Personal Accomplishment:* measures feelings of competence and successful achievement in one's work. ^11^

*Stress* *:* According to Baum, ^19^ stress is an uncomfortable emotional experience accompanied by predictable biochemical, physiological, and behavioral changes.

*Undergraduate dental student:* Undergraduate students enrolled in a dental school.

*Postgraduate dental student:* Students who graduated from a dental school awarding a dental degree, who successfully passed licensure exams and are enrolled in a higher education specialty program. These students are referred to as ‘residents’ when the type of postgraduate program they are enrolled in requires a clinical residency.

*Clinical postgraduate dental specialty programs:* Those programs with a heavy focus on the clinical procedures of a certain branch in dentistry. The postgraduate students enrolled in this type of program are referred to as ‘residents’. These programs typically require residents to diagnose and treat a vast number of patients throughout the duration of the program. They award graduates degrees in the clinical specialties of dentistry. These specialties are: orthodontics, endodontics, periodontics, prosthodontics, pedodontics, oral maxillofacial surgery, oral maxillofacial radiology, oral medicine, operative and restorative dentistry and advanced education in general dentistry (AEGD).

*Non-clinical postgraduate dental specialty programs* *:* Those programs with less focus on direct patient-doctor interactions. They vary in nature and requirements according to the field of study. These programs award degrees in the nonclinical specialties of dentistry, e.g., public health and community dentistry, oral biology and biomaterials etc. This term also potentially includes non-clinical degrees in clinical specialties, e.g., Doctor of Philosophy degrees (PhD) in endodontics.

*Timeline of dental studies in Saudi Arabia:* Undergraduate dental student, intern, licensure exam, dentist, postgraduate dental student.

*Timeline of dental studies in the United States* *:* Pre-dental student, dental student, licensure exams, dentist, postgraduate dental student.

*Timeline of dental studies in the United Kingdom* *:* Undergraduate dental student, foundation dental training, registration for licensure procedure, dentist, postgraduate dental student.

*Note:* For the sake of simplifying, the term ‘undergraduate dental student’ is used in this study to include all dental students pre-graduation from dental school regardless of location.

## Methods

### Ethical approval

This study has been performed in accordance with the Declaration of Helsinki regulations and guidelines. Participation in the study was voluntary and confidential through anonymous, non-identifying survey design. Written informed consent was obtained from all study participants. Approval from the institutional review board (IRB) was obtained on 2/27/2019 with protocol #19-53.

### Participants

Direct communication with the Saudi Arabian Cultural Mission was conducted to identify programs with Saudi Arabian dental residents enrollment. Communication with Saudi Arabian dental residents enrolled in these programs was then initiated by the study representatives in the US and the UK directly whether face-to-face, e-mail, or social media accounts. The target population is Saudi Arabian dental residents enrolled in UK-based and US-based post graduate programs in the following clinical specialties: endodontics, fixed and removable prosthodontics, oral and oromaxillofacial surgery, orthodontics, oral medicine, operative and restorative dentistry, advanced education in general dentistry, oral radiology, and pediatric dentistry and periodontics. These specialties were selected due to their clinical nature with repeated resident-patient interactions. The programs award graduates either a certificate of advanced graduate study (CAGS) or a Master of Science degree (MS, MSc) upon completion. All programs require residents to be proficient in the English language as indicated by their Test of English as a Foreign Language (TOEFL) or International English Language Testing System (IELTS) scores. Non-clinical programs with lowered or no direct patient contact were excluded due to the nature of the study requiring repetitive direct contact with patients to report the burnout experience.

### Sampling procedure

The sampling method used is the non-probability quota sampling technique. Although ideal, a stratified random sample from the population is not feasible *.* Based on a power analysis using the software G*Power, it was determined that to detect a small-medium effect ( *f* ^2^ = .09), a sample size of 158 is needed (α = 0.05, power = .80, # of predictors = 6). Given the main focus of this study being the comparison between residents in two countries (i.e., UK and US), the minimum number of participants desired was 79 participants for each country. The G*Power software can be freely downloaded from http://www.psycho.uni-duesseldorf.de/abteilungen/aap/gpower3/. As it turned out, the sample size obtained was just 87 cases with complete data (from both countries combined) and given the *R* ^2^ values obtained (which were translated into *f* ^2^ values of .23, .02, and .08), the power was .91, .12, and .43 for the regression models predicting EE, DP, and PA, respectively. This limitation to statistical conclusion validity will be noted in the discussion.

### Instrumentation

This study utilized a cross-sectional study design. To address the research questions, the criterion variables are emotional exhaustion (EE), depersonalization (DP), and professional accomplishment (PA); the control variables are hours of work per week, marital status, gender, prior work experience, and sponsorship status; and the predictor variable is country.

The survey starts with an informed consent page followed by the first part of the survey. This first part was created by the author and includes demographic questions regarding program location, age, gender, marital status and family living arrangements. Other questions address specialty program type and length, resident position/year, hours of work per week, sponsorship status and work experience prior to joining the program.

To assess perceived stress and burnout, the second part of the survey includes the English version of the Maslach Burnout Inventory Human Services Survey for Medical Personnel MBI-HSS (MP). It was distributed to participants via a web link. According to Maslach *et al.,* ^11^ sufficient levels of stability estimates and internal-consistency reliability estimates (Cronbach’s alpha) are reported for the three MBI-HS scores from a broad sample of workers in professions related to human-services. Cronbach’s alphas were .90, .79, and .71 for emotional exhaustion, depersonalization, and personal accomplishment, respectively. Multiple studies were cited in which test-retest coefficients for the three scale scores were reported for a variety of samples; for example, over a few weeks (.82, .60; and .80, respectively); three months (.75, .64, and .62, respectively); and up to one year (.60, .54, and .57, respectively). ^11^

The MBI section is the second part of the survey, ^16^ which contains 22 items to assess burnout on the three identified scales: emotional exhaustion, depersonalization and professional accomplishment. Participants will be asked to rank item responses on a seven-point Likert scale where 0 means ‘never’, 1: ‘a few times a year or less’, 2: ‘once a month or less’, 3: ‘a few times a month’, 4: ‘once a week’, 5: ‘a few times a week’, and 6: ‘every day’. The survey was not piloted prior to this study.

### Procedure

Contact was made via e-mail with informal representatives of the Saudi Arabian dental residents in the US and the UK, given their spread out locations from the author, located in Western US and the Northeastern US while the third was in the UK. Moreover, their network connections with Saudi Arabian dental residents who are enrolled in a variety of dental programs in both countries.

The two representatives handed out paper recruitment flyers to residents within both of their schools and sent out emails with the flyers attached to acquaintance residents in other schools. These flyers invited potential participants to provide the representatives with their email addresses for the online survey link to be sent out to. The flyers included assurance statements about the anonymous and voluntary nature of the survey. Once residents responded to the flyer in person or by email by providing their preferred email address, the email addresses were added to a list of recipients on the survey website, SurveyMonkey, and the survey link was forwarded to the list through the website itself. The website used to administer the survey is an online survey tool ( SurveyMonkey.com, Palo Alto, CA, US) allowing for anonymous responses and voluntary participation. Once they received the link *via* email, clicking on it takes the respondent to the consent form page first. Reading and signing the consent by clicking on the appropriate button was mandatory to view the survey questions. Otherwise, the link automatically redirected responders to exit the survey. The anticipated response was 80%. However, due to low participation rate, contact with prominent social media figures in the Saudi dental field on Twitter was initiated. These influencers have many followers who are mostly dental field professionals and students. A web link was distributed in a Twitter post by two social media figures in an attempt to reach out to as many potential respondents as possible. This helped improve response rate significantly. The surveys were available online for a period of three months. Data collection started towards the end of February 2019 and continued until early May 2019. To improve response rate, an incentive of $5 Starbucks™ e-gift cards were to be offered to 20 participants who were going to be chosen randomly. No e-mail addresses were requested or collected in the survey filling process itself. Participants were only given the choice to sign up for the gift cards after filling out the survey where they were asked to provide any e-mail address to send the e-gift card randomly to it. This was done in a separate webpage (PromoSimple.com, Boston, MA, US) which participants were redirected to automatically after completion and submission of the survey. This webpage contained a prompt requesting interested participants to enter an e-mail address to participate in the draw to win the gift card. There was no conflict with confidentiality as there was no connection between the survey responses and the PromoSimple webpage where the e-mail addresses are entered. Moreover, signing up for the gift cards was completely optional and did not require a verified e-mail address. Ultimately, only three participants signed up for the gift cards, which were sent by the author to the provided e-mail addresses without the need for randomization or selection as the number of participants who signed up (3) was below the number required to initiate randomization and selection which was set by the author as 20 interested participants and above. Subsequently, data was downloaded to a Microsoft Excel (2019) spreadsheet and the statistical package for the social sciences (SPSS) software (IBM SPSS Statistics for Windows, version 23 (IBM Corp., Armonk, N.Y., USA) which can be found at ( https://www.ibm.com/sa-en/analytics/spss-statistics-software) was used to run the appropriate statistical analyses.

### Data analysis

The data for this research is available in the Harvard Dataverse repository. ^20^ In order to evaluate the research questions, an α level of 0.05 was employed. Descriptive statistics along with t-tests and multiple regression analysis was used.

The first research question (RQ) involves descriptive statistics including means, medians, and standard deviations which are reported for the three scales of the MBI and compared to existing norms for medical professionals ^21^ using a series of three one-sample t-tests for the three subscales. To address the second RQ, a simultaneous multiple regression analysis was performed for each of the three MBI subscales utilizing the five control variables and the key predictor (country) in the model. The *R* ^2^ was used to gauge the proportion of variation in levels of burnout that the set of variables explains with similar analyses for the other subscales. To address the third RQ, three sequential multiple regression analyses were performed predicting the scores of each subscale separately. In Block 1, the five control variables were entered. In Block 2, the key predictor (country) was entered. The change in *R* ^2^ ( *ΔR* ^2^) and its level of significance was used to answer the research question. The results for both RQ _2_ and RQ _3_ are summarized as shown in Tables 4, 5, and 6.

### Reliability

Reliability was evaluated using Cronbach’s alpha for the three MBI-HSS (MP) scales yielding coefficients of 0.819 for EE, 0.642 for DP, and 0.708 for PA. That the reliability for DP was below .70 suggests a potential threat to statistical conclusion validity for analyses involving depersonalization, a limitation to be noted in the discussion, as well.

## Results

The purpose of the study was to assess the burnout experienced by Saudi Arabian dental residents studying abroad through the means of the MBI survey tool by assessing the three dimensions of burnout; emotional exhaustion (EE), depersonalization (DP), and personal achievement (PA).

The assessment results are compared to other medical professionals results in RQ one. In RQ two and three, subscale scores from each of the three dimensions of the MBI are examined via multiple regression analyses to determine which predictors account for unique variation in the indicators of burnout.

### Demographics

A total of 93 residents responded to the survey. The number of complete responses was 87. A table of descriptive statistics and zero order correlations for all variables is provided as shown in Table 2. Zero order correlations were done to test for correlations between the independent variables. In the descriptive statistics, over two-thirds (68.8%) of respondents were studying in the United States while about one- third (31.2%) of the respondents were studying in the United Kingdom. Male respondents comprised 58.1% of the sample while 41.9% were female. More respondents were married (64.5%) than single (35.5%). Prior work experience, specialty and hours of work per week demographics are shown in Table 3. Most respondents had a few years of prior work experience before beginning their postgraduate programs abroad. Respondents from a wide range of specialties participated in the survey, where Fixed and Removable Prosthodontics residents were the most numerous at 21.5% while Oral Medicine residents were the fewest (2.2%). Most of the sample reported being dual sponsored by the Saudi Cultural Mission and an employer. Over a quarter of the sample reported a high number of work hours per week where they were required to work 51 hours per week in program related activities and requirements.

**Table 2.** Descriptive Statistics and Zero Order Correlations (n = all participants who completed the survey = 87).

	1.	2.	3.	4.	5.	6.	7.	8.	9.	M	SD
1. Country (1 = USA, 0 = UK)	-	-	-	-	-	-	-	-	-	0.68	0.47
2. Hours of Work/week	.15	-	-	-	-		-	-	-	4.63	1.62
3. Sponsorship Status (1= SACM * & Employer, 0 = SACM only)	.16	-.07	-	-	-	-	-	-	-	0.24	0.43
4. Marital Status (1=Married, 0 = Single)	-.05	-.01	-.03	-	-	-	-	-	-	0.64	0.49
5. Prior Work Experience	-.19	-.04	-.07	.16	-	-	-	-	-	2.89	1.45
6. Gender (1 = Female, 0 = Male)	-.12	-.19	-.03	-.22 **	-.13	-	-	-	-	0.42	0.50
7. Emotional Exhaustion (EE)	.27	.23 **	.20	-.08	.04	.19	-	-	-	2.73	1.20
8. Depersonalization (DEP)	.02	-.02	.13	-.00	.01	.07	.55 ***	-	-	1.45	1.16
9. Diminished Personal Accomplishment (PA)	-.02	.11	.58	.010	.05	-.24 **	-.13	-.10	-	4.41	0.96

* SACM = Saudi Arabian Cultural Mission.

** Correlation is significant at the 0.05 level (2-tailed).

*** Correlation is significant at the 0.01 level (2-tailed).

**Table 3.** Demographic data including country, gender, sponsorship status, marital status, and prior work experience of all respondents (n= all participants = 93).

Country	%	*n*	Specialty	%	*n*
UK	31.2	29	Endodontics	12.9	12
US	68.8	64	Fixed and removable prosthodontics	21.5	20
**Gender**	**%**	***n***	Orthodontics	12.9	12
Female	41.9	39	Oral and maxillofacial surgery	3.2	3
Male	58.1	54	Advanced education in general dentistry	9.7	9
**Sponsorship status**	**%**	***n***	Oral medicine	2.2	2
SACM */Employer only or no sponsor	30.1	28	Operative and Restorative dentistry	11.9	11
Dual sponsorship	69.9	65	Periodontics	5.4	5
**Marital status**	**%**	***n***	Pedodontics	8.6	8
Married	64.5	60	Oral and maxillofacial Radiology	4.3	4
Single	35.5	33	Other	7.5	7
**Prior work experience**	**%**	***n***	**Hours of work per week**	**%**	***n***
Less than one year	18.3	17	Less than 10 hours per week	5.4	5
More than one year but less than two	25.8	24	11-20 hours per week	6.5	6
More than two years but less than three	26.9	25	21-30 hours per week	12.9	12
More than three years but less than four	11.8	11	31-40 hours per week	16.1	15
More than four years but less than five	8.6	8	41-50 hours per week	32.3	30
More than five years	8.6	8	51-60 hours per week	10.8	10
			More than 60 hours per week	16.1	15

* SACM = Saudi Arabian Cultural Mission.

### Results regarding RQ one

To address the first research question, as to the level of burnout, one-sample t-test analyses were performed, employing an alpha level of.05 to compare the means of Saudi dental residents to that of the medical profession for the three MBI scales. The means of the medical professionals (2.466, 1.424, and 4.566 for EE, DP, and PA, respectively) were obtained from the MBI manual. ^11^ There is evidence to suggest a statistically significant difference between the means of Saudi Arabian dental residents ( *M* = 2.73, *SD* = 1.20) and the medical professionals population means in EE ( *p* = .043). Specifically, we are 95% confident that the dental residents are at least .009 and at most.52 points higher in emotional exhaustion. However, there was insufficient evidence to suggest a statistically significant difference in DP ( *p* = .860) and PA ( *p* = .510), based on the dental residents’ sample statistics ( *M* = 1.45, *SD* = 1.16) and ( *M* = 4.41, *SD* = .96) respectively as shown in Table 2.

### Results regarding RQ two and three

Multiple regression analyses were conducted to address research questions two and three regarding the proportion of variation accounted for by the set of six predictors and which predictors account for unique variation in the levels of burnout. Tables 4, 5 and 6 summarize the results of analyses for the three subscales, respectively. In these tables, Block 1 consists of independent variables (hours worked per week, sponsorship status, gender, marital status and years of prior work experience) and Block 2 contains the dependent variable (country).

**Table 4.** Sequential multiple regression results predicting Maslach Burnout Inventory (MBI) emotional exhaustion subscale scores.

	*b*	*SE _b_*	*β*	*t*	*p*	*R*2	*ΔR* ^2^
Block 1						.168	.168
Hours worked per week	.229	.077	.311	2.993	.004		
Sponsorship (1 = yes)	.712	.286	.256	2.491	.015		
Gender (1 = female)	.550	.263	.226	2.091	.040		
Marital status (1 = married, 0 = single)	-.097	.260	-.039	-.374	.709		
Years of prior work experience	.065	.087	.079	.748	.457		
Block 2						.188	.020
Country (1 = US, 0 = UK)	-.383	.274	-.150	-1.398	.166		

Notes: 1. Full Model: *F*(6, 80) = 3.087, *p*= .009, *R* ^2^ = .188. 2. Change: *F*(1, 80) = 1.954, *p* = .166, *R* ^2^ = .020.

**Table 5.** Sequential multiple regression results predicting Maslach Burnout Inventory (MBI) depersonalization subscale scores.

	*b*	*SE _b_*	*β*	*t*	*p*	*R*2	*ΔR* ^2^
Block 1						.021	.021
Hours worked per week	.002	.081	.002	.022	.983		
Sponsorship (1 = yes)	.343	.304	.127	1.129	.262		
Gender (1 = female)	.187	.280	.079	.668	.506		
Marital status (1 = married, 0 = single)	.036	.276	.015	.129	.898		
Years of prior work experience	.020	.092	.026	.222	.825		
Block 2						.022	.000
Country (1 = US, 0 = UK)	.057	.291	.023	.197	.844		

Notes: 1. Full Model: *F*(6, 80) = .297, *p* = .937, *R* ^2^ = .022. 2. Change: *F*(1, 80) = 0.039, *p* = .844, *R* ^2^ = .000.

**Table 6.** Sequential multiple regression results predicting Maslach Burnout Inventory (MBI) personal achievement subscale scores.

	*b*	*SE _b_*	*β*	*t*	*p*	*R*2	*ΔR* ^2^
Block 1						.070	.070
Hours worked per week	.050	.065	.085	.772	.443		
Sponsorship (1 = yes)	.162	.244	.073	.663	.509		
Gender (1 = female)	-.490	.225	-.252	-2.181	.032		
Marital status (1 = married, 0 = single)	-.101	.222	-.051	-.457	.649		
Years of prior work experience	.013	.074	.019	.172	.864		
Block 2						.078	.008
Country (1 = US, 0 = UK)	-.193	.234	-.095	-.828	.410		

Notes: 1. Full Model: *F*(6, 80) = 1.128, *p* = .354, *R* ^2^ = .078. 2. Change: *F*(1, 80) = .686, *p* = .410, *R* ^2^ = .008.

*Emotional exhaustion*

In Table 4, the multiple regression model for EE with all six predictors was statistically significant and explained 18.8% of the variation, *F*(6, 80) = 3.087, *p* = .009, *R* ^2^ = .188. Whereas the set of five variables in the first block accounted for 16.8% of the variance, knowing the country in which the student was a dental resident explained an additional 2.0% of the variance in EE. This increase, however, was not statistically significant, *F*(1, 80) = 1.954, *p* = .166. Three of the predictors accounted for unique variation in levels of EE. The unstandardized regression coefficient for hours worked per week, *b* = .229, *t* (80) = 2.993, *p* = .004, indicates that for each additional unit increase of the predictor (10 hours increments), EE scores increased by .229 points, controlling for other predictors. Of more interest was the coefficient associated with Sponsorship, *b* = .712, *t* (80) = 2.491, *p* = .015, where being sponsored is associated with an increase of .712 points on the EE scale while controlling for other predictors.

Once other variables are taken into account, gender was a statistically significant predictor for the EE scale as well. On average, the EE scores of females (coded 1) were .550 points higher than males (coded 0), *b* = .550, *t* (80) = 2.091, *p* = 040. Those studying in the US (coded 1) had lower EE scores, on average, than those studying in the UK (coded 0), but the difference was not statistically significant, *b* = -.383, *p* = .166.

*Depersonalization*

In Table 5, the multiple regression model for depersonalization (DP), the set of six predictors was statistically significant but explained just 2.2% of the variation, *F*(6, 80) = .297, *p* = .937, *R* ^2^ = .022. Whereas the set of five variables in the first block accounted for 2.1% of the variance, knowing the country in which the student was a dental resident explained less than 1% more of the variance in DP. This increase was not statistically significant, *F*(1, 80) = 0.039, *p* = .844. None of the predictors accounted for unique variation in levels of DP. Those studying in the US (coded 1) had higher DP scores, on average, than those studying in the UK (coded 0), but the difference was not statistically significant, *b* = .057, *p* = .844.

*Personal accomplishment*

In Table 6, the multiple regression model for personal accomplishment (PA) with all six predictors was not statistically significant and explained 7.8% of the variation, *F*(6, 80) = 1.128, *p* = .354, *R* ^2^ = .078. Whereas the set of five variables in the first block accounted for 7.0% of the variance, knowing the country in which the student was a dental resident explained less than 1% of the variance in PA. This increase, then, was not statistically significant, *F*(1, 80) = .686, *p* = .410. Gender was the only predictor that accounted for unique variation in levels of PA. On average, the PA scores of females (coded 1) were.490 points lower than males (coded 0), *b* = -.490, *t* (80) = -2.181, *p* = .032. Those studying in the US (coded 1) had lower PA scores, on average, than those studying in the UK (coded 0), but the difference was not statistically significant, *b* = -.193, *p* = .410.

## Discussion

The findings of this study aligned with those of previous research in terms of burnout prevalence in dental postgraduate students. Mandava *et al.,* ^13^ and Divaris *et al.,* ^12^ reported a similar positive correlation between stress and burnout by employing different methodologies. However, some results suggested new information when compared with previous research findings. Mandava *et al.,* ^13^ found no significant differences between males and females in reporting burnout.

Multiple studies suggested higher emotional exhaustion, depersonalization and diminished personal achievement in physicians ^22^ and health care staff. ^23^ Medical professional MBI scales means were used to compare burnout reporting of dental residents to the general medical professionals population. The sample mean for the EE MBI scale was significantly different from the medical professionals population mean provided by the MBI manual. The sample means were significantly higher in EE compared to the medical professional means. This suggests that Saudi Arabian dental residents experience high emotional exhaustion when compared to other medical professionals. The DP and PA scales means were not found to be significantly different between the two.

In the multiple regression analysis, the EE model was found to vary significantly according to hours of work per week, sponsorship status, and gender. This suggests that increased workload contributes to higher emotional exhaustion. Interestingly, dual income residents from a sponsor job and the SACM experienced more emotional exhaustion than their SACM-only single income or self-sponsored counterparts. This could be explained by the added pressure from employers on residents to meet multiple requirements and achievements before returning to the workforce. Moreover, it is suggested that females experienced more emotional exhaustion and less sense of personal achievement than their male counterparts. This aligns with a study by Minamizono *et al.,* ^24^ suggesting that female nurses in Japan reported higher stress and subsequent intention to leave careers due to assigned gender roles and socio-cultural pressures. Balancing work and family life could potentially contribute to added pressures on females in health care. Interestingly, marital status and prior work experience did not explain any variance or difference in the analyses. This differs from findings by Al-Shayea ^15^ where married students were found to report less stress and burnout compared to their single counterparts.

Burnout is a serious problem facing healthcare professionals. International students are at an even higher risk potentially due to change in social and professional environment, cultural differences and financial limitations. The following presents some suggestions for problems facing healthcare professionals.

For dental residents, it is recommended to find ways to reduce stress and sequentially burnout by time management measures, relaxation and leisure activities. Residents need to be aware of the symptoms of burnout and educate themselves about the signs and how to combat them as early as possible. They should not be afraid to seek help or guidance from their supervisors or program directors.

Residency programs can improve residents’ experiences by providing seminars to educate residents about burnout and plan regular leisurely activities with possible credits towards their degrees to entice residents into participation. Female residents were found to experience higher burnout in this study sample. This was supported by results of another study as well. ^24^ Therefore, females should be considered a critical group to be further encouraged to de-stress and express their perception of personal achievements with faculty or counselors as a part of their official programs. A required psychology course can be offered as a part of the dental residency program to allow dental residents to explore a variety of psychological issues that could affect their careers, their patients and patient care. Another recommendation for dental programs would be to provide online counseling for health care specialties students. This option provides convenience for these students who often have rotations in multiple locations (e.g. clinics, hospitals and campuses) so that they can access psychological care online anywhere.

The Saudi Arabian sponsors investment in the medical and dental health care professionals can benefit from providing these individuals with a good experience during their studies abroad. Alleviating burnout in residents can be achieved by simplifying the necessary procedures and paperwork required to maintain sponsorship. Saudi Arabian academic advisors can play an integral role in providing mental health support to those in need of it. These measures should enhance their academic experience and subsequently their professional performance once they return to practice in Saudi Arabia. It can also help in retaining health care professionals and their relationships with co-workers and staff to enhance the health care services they provide to the Saudi Arabian patient population.

Burnout is a multifactorial issue facing healthcare specialty students. It can be reduced through the efforts of all the stakeholders involved in the students’ education process. It is imperative to further explore this phenomenon and employ a multitude of ways to overcome this issue to aim for better mental health care and psychological stability for health care professionals leading to elevated performance and optimum experiences for patients seeking their services and expertise.

### Assumptions and limitations

Statistical assumptions include those underlying the use of multiple regression analysis such as: normality, linearity, homoscedasticity, and independence of the error terms. Methodological limitations are self-reporting and recall bias. Self-reporting assumes complete transparency in responding to the survey. However, the effect of this assumption is assumed to have been minimized by the anonymous and voluntary nature of this study. Moreover, responses from residents in different dental specialty programs limit the equal distribution of subjects across the specialty type category. This study is also limited by the concurrent political circumstances of the program countries and Saudi Arabia. It is also important to note that generalizability or external validity is limited to the characteristics of the participants. Furthermore, some dental specialties were not included in this study due to the nature of these programs being only indirectly interactive with patients, e.g., oral histology and oral pathology.

Reliability testing of the MBI scales revealed sufficient internal consistency for EE and PA but not for DP. This can be related to the number of items in the scale. DP has five items only while EE and PA have nine and eight items respectively. This limitation has likely affected the multiple regression results pertaining to DP where none of the individual predictors was statistically significant. This could also be explained by another limitation this study faced where the number of complete responses were insufficient to detect subtle effects. Increasing the incentive promised to participants or contacting residency program directors requesting recruitment of their residents in a formal way where they set aside time for residents to complete the survey during working hours could have increased the response rate. However, due to time and financial constraints and the researcher’s limited social network in dental schools in the US and the UK, these methods were not feasible. There was a difficulty in reaching out to all residents due to variation in physical location between different countries, schools and specialties. This results in limited statistical power to the analysis and poses a threat to statistical conclusion validity.

### Suggestions for further research

More investigation is needed to evaluate the differences in reported burnout in the excluded specialties and across various program types, e.g., fellowship and PhD programs, to broaden our understanding of this phenomenon and inform future residents who are opting to pursue postgraduate studies. Although this is a comparative study, it is imperative to adopt a longitudinal study design to allow for causal inferences in understanding how burnout progresses over time in health care professionals and the effect it could have on patient care and medical errors. Cultural and social norms affecting female professionals should be explored to ensure better awareness in this critically affected subgroup, in particular. Moreover, qualitative study designs utilizing open ended questions could be used to elicit, for example, participants’ experience as a resident, including their feelings related to burnout and the expectations they perceive their sponsors to have for them during their programs.

## Conclusion

The literature suggests that burnout is a common problem among dental postgraduate students. This study found that Saudi dental postgraduate students experience higher emotional exhaustion when compared to other professionals in the medical field. Female residents are more likely to report experiencing emotional exhaustion compared to their male colleagues. Further investigations are needed to study burnout effects in dental postgraduate students throughout the duration of their studies.

## Data availability

### Underlying data

Harvard Dataverse: Asiri, Amal, 2022, “Saudi Arabian Students in Postgraduate Dental Programs: Investigating Factors Associated with Burnout”, https://doi.org/10.7910/DVN/NTBYPX. ^20^

This project contains the following underlying data:

• Excel Data File with Legend. (Anonymized survey responses for demographic and MBI sections)
• SPSS Data File. (survey responses tabulations)

Data are available under the terms of the Creative Commons Zero “No rights reserved” data waiver (CC0 1.0 Public domain dedication).

### Extended data

Harvard Dataverse: Asiri, Amal, 2022, “Saudi Arabian Students in Postgraduate Dental Programs: Investigating Factors Associated with Burnout”, https://doi.org/10.7910/DVN/NTBYPX. ^20^

This project contains the following extended data:

• Demographic part of the survey (blank English copy of the demographic section of the survey).
• Participants Recruitment Flyer.

Data are available under the terms of the Creative Commons Zero “No rights reserved” data waiver (CC0 1.0 Public domain dedication).
