# Supplementary material for: Saudi Arabian students in postgraduate dental programs: Investigating factors associated with burnout
Source: F1000Res. 2022 Nov 17;11:705. Originally published 2022 Jun 27. [Version 2] doi: 10.12688/f1000research.111000.2 (PMC13554729; doi:10.12688/f1000research.111000.2)
Supplement: Peer review history

# Reviewer report: Reviewer response for version 2

**DOI:** 10.5256/f1000research.141134.r219516

**Published:** 2026-07-21

**Authors:** Lalitha Rani Chellappa

**Affiliations:** 1 Saveetha Institute of Medical and Technical Sciences (SIMATS), Saveetha University, Chennai, India

**1. Limited sample size and statistical power**

 The planned sample size was 158, whereas only 87 complete responses were obtained. This substantially limits statistical power, particularly for the depersonalization and personal accomplishment regression models. 

**2. Sampling method and external validity**

 The study employed non-probability quota sampling through personal networks and social media recruitment. Although understandable, this introduces potential selection bias and limits the generalisability of the findings

**3. Regression assumptions**

 Although the manuscript states that multiple regression assumptions were considered, the results do not report diagnostic testing such as residual normality, multicollinearity, homoscedasticity or influential observations. Reporting these diagnostics would strengthen confidence in the regression analyses.

**4. Potential confounding variables**

 Several important predictors of burnout were not included, including year of residency, specialty type, financial stress, perceived social support, English language proficiency, and previous mental health history. These should be acknowledged as potential residual confounders.

Is the work clearly and accurately presented and does it cite the current literature?

Yes

If applicable, is the statistical analysis and its interpretation appropriate?

Partly

Are all the source data underlying the results available to ensure full reproducibility?

Yes

Is the study design appropriate and is the work technically sound?

Yes

Are the conclusions drawn adequately supported by the results?

Yes

Are sufficient details of methods and analysis provided to allow replication by others?

Partly

Reviewer Expertise:

public health, epidemiology, systematic reviews, and biostatistics

I confirm that I have read this submission and believe that I have an appropriate level of expertise to confirm that it is of an acceptable scientific standard, however I have significant reservations, as outlined above.

# Reviewer report: Reviewer response for version 2

**DOI:** 10.5256/f1000research.141134.r299504

**Published:** 2024-07-08

**Authors:** Aidos Bolatov

**Affiliations:** 1 Medical School, Shenzhen University (Ringgold ID: 47890), Shenzhen, Guangdong, China; 2 Astana Medical University, Astana, Kazakhstan

The study investigates burnout among Saudi Arabian dental residents in the US and UK. Using the Maslach Burnout Inventory (MBI), the research identifies emotional exhaustion (EE), depersonalization (DEP), and personal accomplishment (PA) among 87 respondents. Factors such as work hours, sponsorship, and gender predict EE, while only gender predicts PA. DEP is not predicted by any variables. The study highlights high burnout levels in Saudi Arabian dental residents, particularly among females, and underscores the need for institutional support to mitigate burnout.

 1. The Introduction section talks a lot about stress, whereas the study itself analyzes only burnout. What was the need to conduct a fairly large review of the literature on the topic of stress? After all, the results of the study cover only such a parameter as burnout, measured by the MBI scale.

 2. Only in the Introduction section the 3-component structure of the MBI burnout scale is mentioned 5-6 times. There is no need to explain the scale structure in one text so many times.

 3. Definition of terms – in my opinion, it is better to move this section to Supplementary Materials. There is no need to prescribe them in the text of the article.

 4. Ethical approval – write the full name of the institution that issued you the ethical approval.

 5. During what period was the survey conducted?

 6. The discussion could be strengthened by exploring additional factors that might contribute to burnout, such as cultural adaptation and institutional support.

Is the work clearly and accurately presented and does it cite the current literature?

Partly

If applicable, is the statistical analysis and its interpretation appropriate?

Yes

Are all the source data underlying the results available to ensure full reproducibility?

Yes

Is the study design appropriate and is the work technically sound?

Yes

Are the conclusions drawn adequately supported by the results?

Partly

Are sufficient details of methods and analysis provided to allow replication by others?

Partly

Reviewer Expertise:

Mental health, Health Psychology

I confirm that I have read this submission and believe that I have an appropriate level of expertise to confirm that it is of an acceptable scientific standard, however I have significant reservations, as outlined above.

# Reviewer report: Reviewer response for version 2

**DOI:** 10.5256/f1000research.141134.r156028

**Published:** 2022-12-01

**Authors:** S Kimberly Haslam

**Affiliations:** 1 School of Dental Hygiene, Faculty of Dentistry, Dalhousie University, Halifax, NS, Canada

Sorry for the delay in reviewing this as I told the editorial team, I was extremely sick with Covid. 

 You have addressed all of my concerns. The paper as a whole has been greatly improved. Well done. 

 I would have suggested that the definition of terms, be an appendix.  But the way you have it written is fine.

Is the work clearly and accurately presented and does it cite the current literature?

Yes

If applicable, is the statistical analysis and its interpretation appropriate?

I cannot comment. A qualified statistician is required.

Are all the source data underlying the results available to ensure full reproducibility?

Yes

Is the study design appropriate and is the work technically sound?

Yes

Are the conclusions drawn adequately supported by the results?

Partly

Are sufficient details of methods and analysis provided to allow replication by others?

Yes

Reviewer Expertise:

Burnout in dental hygiene professionals and oral health students (dental hygiene and dentistry)

I confirm that I have read this submission and believe that I have an appropriate level of expertise to confirm that it is of an acceptable scientific standard.

# Reviewer report: Reviewer response for version 1

**DOI:** 10.5256/f1000research.122666.r153979

**Published:** 2022-11-02

**Authors:** S Kimberly Haslam

**Affiliations:** 1 School of Dental Hygiene, Faculty of Dentistry, Dalhousie University, Halifax, NS, Canada

Usually depersonalization is abbreviated DP not DEP.

**Introduction:**

 Remove memorization to understanding or another verb. I hope dental students do not just memorize the material.

*Persistent exposure to stress can lead to burnout, which involves work or school-related exhaustion and disengagement.4*- should include reduced personal accomplishment. A better reference would be one of the Maslach articles. 

 Unless it is necessary, the information from other studies is a bit long. Do not have to state response rate, how many people were enrolled in the study etc. Instead just the main findings.  

**Definition of Terms**

 Should use the definition for the MBI-HSS MP (which included medical personal). Also include definitions for EE, DP and PP - If possible I would suggest a glossary at the end of the paper with definitions. Defining Burnout, EE, DP and PA in the introduction would be preferable.  

 Post-graduate students should have the disciplines listed so it does not need to be repeated in the methods 

**Methods:**

 Did not state the methodology used: Cross sectional study perhaps?

**Instrumentation**

*Multiple studies were cited in which test-retest coefficients for the three scale scores -* this was not cited?

**Procedure**

 What happened to the list of emails after they were uploaded on Survey Monkey? Drawing the emails from a hat, does that break confidentiality?

**Data Analysis**

 The Table should be in the results - not 100% sure why you did a zero order correlation on this section as it is just demographics.

**Results:**

 I thought your participants were dental residents, you include medical professional population. Where did you get these results from? If you did not survey the medical population then this section should be in the discussion. 

 I looked but could not find a description for Block 1 & Block 2

**Discussion:**

 Maslach studies the 3 domains of burnout, in their research they do not give a total burnout score. Please be aware the EE is not burnout - it is one of the domains of burnout. Some researchers do calculate a burnout score "An alternative approach considers individuals to have burnout if they have a high EE score plus ***either*** a high DP score ***or*** a low PA score (PA score less than 33)"  

 Therefore, I would make it clear in this sentence that you are talking about EE: *"dental residents experience high burnout due to emotional exhaustion when compared to other medical professionals." *Should read High levels of EE  (not burnout) when compared...

 This is throughout the discussion, please be specific to the domains. 

**Limitations**

 I have always been taught not to use contractions in professional papers 'It's"

 There are limitations to survey based research - such as recall bias. 

**Conclusion**

 The first paragraph is not a conclusion from your study. Could state something about the prevalence of burnout in the dental residents instead. 

 A conclusion should not contain new information. It is a recap of the main points. The conclusion in the abstract is more applicable to the topic. Here you are introducing ways to reduce stress, which is great, but it should be in the discussion not the conclusion.

 Also there was not a research question on this topic. All this is based on previous research, yet there is limited citations. I would suggest rewriting the conclusion.  

**Citations** 

 # 15 this is a link to an individual report on the Maslach survey.  .

Is the work clearly and accurately presented and does it cite the current literature?

Yes

If applicable, is the statistical analysis and its interpretation appropriate?

I cannot comment. A qualified statistician is required.

Are all the source data underlying the results available to ensure full reproducibility?

Yes

Is the study design appropriate and is the work technically sound?

Yes

Are the conclusions drawn adequately supported by the results?

Partly

Are sufficient details of methods and analysis provided to allow replication by others?

Yes

Reviewer Expertise:

Burnout in dental hygiene professionals and oral health students (dental hygiene and dentistry)

I confirm that I have read this submission and believe that I have an appropriate level of expertise to confirm that it is of an acceptable scientific standard, however I have significant reservations, as outlined above.
